# Inorganic Nanoparticles Containing Plant‐Derived Compounds for Kidney Treatment

**DOI:** 10.1155/bca/8743377

**Published:** 2025-12-17

**Authors:** Michel Stéphane Heya, David Gilberto García-Hernández, Romario García-Ponce, Donald Fernandes

**Affiliations:** ^1^ Research Center in Nutrition and Public Health, School of Public Health and Nutrition, Autonomous University of Nuevo León, San Nicolás de los Garza, Nuevo León, Mexico, uanl.mx; ^2^ Department of Chemistry, Faculty of Biological Sciences, Autonomous University of Nuevo León, San Nicolás de los Garza, Nuevo León, Mexico, uanl.mx; ^3^ Department of Chemistry and Biology, Toronto Metropolitan University, Toronto, Canada, ryerson.ca

**Keywords:** antioxidants, inorganic nanoparticles, nephroprotection, oxidative stress

## Abstract

Several studies have shown that many kidney diseases are associated with oxidative stress caused by factors such as changes in diet, environmental pollution, and the excessive use of medications, which contribute to cellular damage in the kidneys. This pathology, whose prevalence is increasing, presents a significant challenge for current medicine due to the multiple physiological barriers that limit the effectiveness of conventional treatments. In response to this issue, inorganic nanoparticles synthesized through green methods, using derivatives from medicinal plants as antioxidants (such as flavonoids and polyphenols, among others), have emerged as a promising therapeutic alternative. This approach not only avoids the use of toxic chemical reagents but also allows for the design of nanoparticles with specific physicochemical properties, such as size, charge, and shape, which facilitate their passage through the digestive system, evasion of the immune system, and targeted delivery to renal tissue. The objective of this study is to analyze the potential of inorganic nanoparticles as an innovative therapeutic strategy for the treatment and prevention of kidney diseases, leveraging their ability to protect the kidneys from oxidative damage caused by reactive oxygen species.

## 1. Introduction

Since their first detection in a biological system in the middle of the 1950s, free radicals have become important in biomedical science. Many reactive species (RS) are generated in vivo under physiological concentrations and act as mediators in a variety of physiological processes. Intensified production of RS, whether from endogenous or exogenous sources, may also be involved in the development of pathological changes. RS are implicated in various human disease states like neoplastic, neurodegenerative, ophthalmologic, diabetes‐related, and atherosclerotic diseases, among others. Kidney diseases have also been associated with oxidative stress and RS, adding to the relevance of studying these mechanisms in renal pathology [1].

The kidneys are important organs in human physiology, which are involved in water and fluid balance, the maintenance of blood pressure, the production of erythrocytes and bone density, hormonal balance, and the removal of the nitrogen‐bearing and other waste products. Chronic kidney disease (CKD) is defined as a gradual decrease in kidney function, while acute kidney injury (AKI) is a sudden loss of kidney function. Cases of CKD and AKI are increasing globally and are of significant public health concern. By 2040, CKD is projected to become the fifth leading cause of death worldwide. Moreover, kidney diseases have been reported as risk factors for severe COVID‐19 outcomes [2]. Nephrotoxicity is one of the most frequent kidney problems, which happens whenever the body is purified of a drug or toxin. Nephrotoxicity may be “true” renal disease or failure that develops either directly or indirectly because of exposure to medicaments and environmental or industrial chemicals. Several variables are affected due to toxic injury from the use of indigenous medicines, such as urine pH, blood flow, endothelial surface area, metabolic activity, active uptake by tubular cells, and medullary interstitial concentration [3].

The antioxidant system is composed of many reactive oxygen species (ROS) scavenging enzymes. Glutathione (GSH) and the thioredoxin‐2 system constitute the major thiol antioxidant systems in mitochondria, which require NADPH/NADP^+^ reducing power. GSH is a major endogenous antioxidant produced by cells. Evidence suggests that oxidative damage plays a central role in the pathogenesis of diabetic nephropathy. ROS are also involved in the development of oxidative stress and generation of several inflammatory cytokines that cause damage to proteins, lipids, and nuclear DNA [4]. The treatment of kidney diseases faces considerable challenges due to multiple physiological barriers that reduce the effectiveness of conventional therapies [5, 6]. Factors such as poor bioavailability, immune system clearance, and inability to reach renal tissues limit the success of current interventions [7]. Nephrotoxicity from traditional drugs adds another layer of complexity to therapy design.

Inorganic nanoparticles containing plant‐derived antioxidants have emerged as a promising alternative in renal medicine, offering promising nephroprotective properties. Green synthesis utilizes phytocompounds such as polyphenols, flavonoids, and terpenes, which act as reducing and stabilizing agents in nanoparticle synthesis, reducing the need for toxic chemical reagents [8]. For example, several studies have demonstrated that silver nanoparticles (AgNPs) synthesized with *Alpinia officinarum* extract exhibit antioxidant and anti‐inflammatory properties, mitigating cisplatin‐induced nephrotoxicity in *in vivo* models [9]. Additionally, zinc oxide nanoparticles obtained from *Solanum torvum* extracts have shown beneficial effects on renal function without evidence of significant toxicity in rat studies [10]. These advances suggest that the combination of nanomedicine and phytotherapy may offer safer and more sustainable treatment options for renal protection [5, 6, 11]. In this context, nanotechnology has emerged as an innovative strategy to overcome these obstacles, enabling the design of nanoparticles with specific physicochemical properties (e.g., size, surface charge, shape) that facilitate their passage through the digestive system and evasion of the immune system [12]. Furthermore, inorganic nanoparticles, due to their biocompatibility and ability to be functionalized with natural compounds, show considerable potential for exerting direct therapeutic effects in renal tissue, especially against damage caused by ROS [13]. This approach opens new possibilities for the development of more effective, safe, and targeted therapies in the treatment and prevention of renal pathologies.

In accordance with the above, the aim of this study is to investigate how inorganic nanoparticles, designed to overcome the physiological barriers of the body, can be used as an innovative therapeutic strategy in the treatment and prevention of kidney diseases. Their ability to protect the kidney from damage caused by ROS and oxidative stress is considered a key factor in preventing the development of conditions such as nephrotoxicity.

## 2. Oxidative Stress Induction Factors in the Kidney

**Figure 1:**
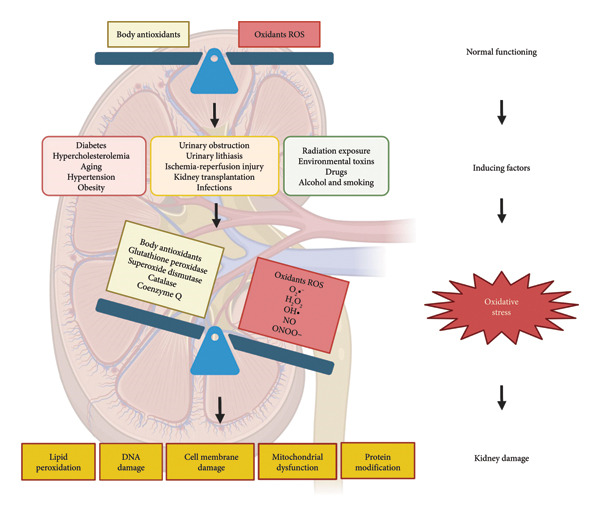
Effects on the kidney of antioxidant deficiency by oxidative stress‐inducing factors.

The kidney is an organ that is particularly vulnerable to oxidative damage due to its high metabolic activity and its role in filtering and eliminating toxins. Additionally, its composition of lipids contains a high concentration of long‐chain polyunsaturated fatty acids, making it prone to lipid peroxidation [14, 15]. Oxidative stress in the kidneys occurs when there is an imbalance between the production of free radicals and ROS and depends on the capacity of antioxidant systems to neutralize them. This excess of free radicals leads to lipid peroxidation, DNA and cell membrane damage, mitochondrial dysfunction, and protein modifications, thereby contributing to the deterioration of renal function (Figure 1).

**Figure 2:**
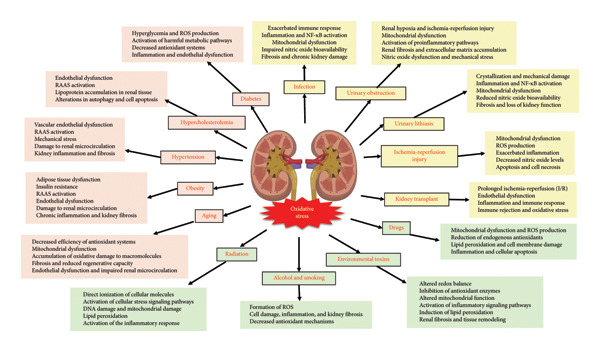
Factors and mechanisms of oxidative stress in the kidney.

Under normal physiological conditions, the ROS produced are completely inactivated by cellular and extracellular defense mechanisms. However, under certain pathological conditions or due to various exogenous factors, several mechanisms are activated that lead to a reduction in the antioxidant defense system, resulting in increased ROS activity and oxidative stress, ultimately causing tissue damage (Figure 2) [14–26].

## 3. Natural Antioxidants With Nephroprotective Potential

Natural products have played a major role in the development of therapeutic agents for the treatment of human diseases. As well as being used for drugs, natural products are also important for the prevention of toxicities and organ damage. For example, in the prevention of cancer, phytochemicals have been administered. The protective activities of natural products have been reported due to their antioxidant and anti‐inflammatory roles. Naturally occurring substances have been widely used for nephroprotective activities. Among the naturally occurring substances, higher plant‐derived compounds have been reported to contribute to nephroprotective activities. Curcumin is a representative compound undergoing clinical evaluation for its nephroprotective activity [27]. Polyphenolic compounds, especially, have been effective in preventing renal dysfunction because of their radical scavenging and antioxidant activities [28, 29]. Plants have been used for medications all over the world for thousands of years. As discussed in the World Health Organization bulletin, an estimated 80% of people in developing countries are still greatly dependent on plant‐based medications for addressing their principal healthcare issues. Seeds and herbs are also used as medicine for diseases. Their use was improved in many areas due to their safety and low side effects as compared with semi‐synthetic and synthetic drugs [3]. Plants are easily available, and consequently, most of the earlier discoveries of the medicinal and bioactive principles for human use were from plant life. These organisms are a good source of bioactive compounds, which often have defined medicinal properties and protective functions, including nephroprotection [30]. Some secondary metabolites are described in Table 1 with their nephroprotective activity.

**Table 1:** The structure and biological activity of a few important secondary metabolites.

Group	Name	Structure	Biological activity	References
Anthraquinones	Rhein		Reduced drug‐induced oxidative damage	[31, 32]

Alkaloids	Ligustrazine		Attenuated tubulointerstitial fibrosis	[33, 34]
	Berberine		Attenuated inflammatory responses and improved glomerular pathological changes in vivo in murine models with diabetes induced	[35, 36]
	Leonurine		Could attenuate renal fibrosis	[37, 38]

Terpenoids	Crocin		Could decrease high levels of serum creatinine, blood urea nitrogen, inflammation, and tubular desquamation in diabetic kidneys with reduced histological damage, oxidative stress, apoptosis, and improved renal function	[39, 40]

Flavonoids/polyphenols	Kaempferol		Reduced serum creatinine, urea nitrogen, KIM‐1, and suppressed NF‐kappaB p65 and COX‐2 in cisplatin‐induced rats	[41, 42]
	Chrysin		May reduce accumulation of myofibroblast‐like cells and matrix proteins in diabetic kidneys	[43, 44]
	Quercetin		Could protect the impairment of kidney functions, protect from oxidative injury, and improve renal function	[45–47]
	Scutellarin		Exerted anti‐inflammatory, hypoglycemic, and renal protective effects	[48–50]
	Resveratrol		Anti‐inflammatory and histoprotective effects in diabetic kidneys could prevent cisplatin‐induced nephrotoxicity and diabetic renal fibrosis	[51–54]
	Naringin		Protective effect against methotrexate (MTX)‐induced nephrotoxicity in experimental animals	[55–57]
	Silymarin		Protective effect against MTX‐induced nephrotoxicity in experimental animals	[57–59]
	Salvianolic acid		Anti‐inflammatory, antiapoptotic, antioxidative, and antithrombotic properties	[60–62]

Diterpene glycosides	Stevioside		Can inhibit the gasdermin D (GSDMD) signaling pathway and could modulate oxidative damage in the liver and kidney of high‐fat/low‐streptozocin diabetic rats	[63–65]

Various plant extracts and their bioactive compounds have been utilized in the treatment of kidney‐related diseases. Notable examples include garlic (*Allium sativum* L.), Chinese skullcap (*Scutellaria baicalensis*), prickly pear cactus (*Opuntia stricta*), Swiss chard (*Beta vulgaris*), Chinese goldthread (*Rhizoma coptidis*), turmeric (*Curcuma longa*), and green tea (*Camellia sinensis*), among others. The therapeutic potential of these phytochemicals has been supported by pilot and clinical studies [66]. In recent years, attention has focused on purifying and identifying the active components of these nephroprotective plant extracts and understanding how these natural compounds might offer protection against nephrotoxicity and kidney injury. Natural substances might have strong preventive properties. Such data exploring this potential property are lacking in preclinical and clinical settings. Therefore, studies on using natural substances in the prevention of diabetes mellitus complications and diabetic nephropathy are important.

## 4. Inorganic Nanoparticles as a System to Overcome Physiological Barriers to Reach the Kidney

**Figure 3:**
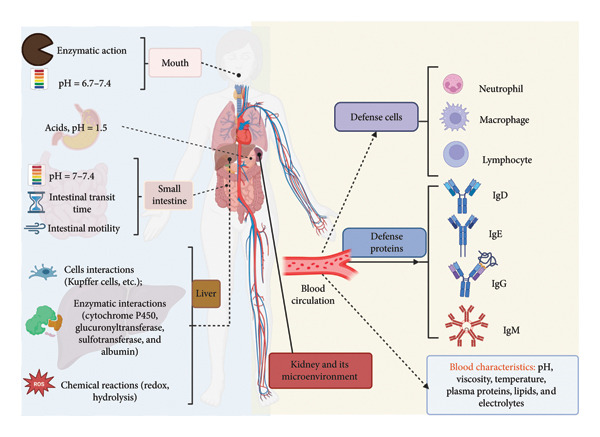
Physiological barriers for drugs administered orally or systemically targeting the kidneys. Orally administered drugs must cross both exogenous and endogenous barriers to reach the kidneys, whereas intravenously administered drugs only need to cross systemic barriers. These barriers can significantly impact the pharmacokinetics and pharmacodynamics of drugs, particularly when they are synthesized without a specific drug‐target strategy.

The kidney plays a crucial role in maintaining homeostasis, and its dysfunction can have serious consequences, such as hypertension and inflammatory diseases. Due to factors such as diet, environmental pollution, and exposure to pharmaceuticals, kidney disease has become an epidemic. Current estimates suggest that approximately 850 million people suffer from some form of kidney problem. The treatment of renal conditions is costly and depletes public health resources, with a high rate of therapeutic failure due to physiological limitations in humans that can affect pharmacological efficacy. Recent studies have identified various barriers that limit pharmacological efficacy in the treatment of renal conditions. According to Thomas and Weber, these barriers include physiological barriers within the individual (Figure 3), such as exogenous barriers (i.e., from the mouth to the liver) and endogenous barriers (i.e., from the liver to the kidney), as well as barriers to kidney permeability and individual heterogeneities within a population [67]. In this section, we will focus on pharmacological limitations at the individual level and within the renal microenvironment, with the aim of better understanding the challenges in the treatment of renal conditions and exploring potential solutions to improve therapeutic efficacy.

The physiological barriers that limit the success of therapies for curing or preventing kidney diseases are complex and multifaceted. These barriers can be classified into two main categories, depending on the method of drug administration. In the case of oral administration, medications may be affected by various exogenous barriers that range from the mouth to the liver. These barriers include digestive enzymes, such as amylases and cytochrome P450, which can break down or alter the medications. Additionally, pH changes in the gastrointestinal tract, which can range from 1.5 to 7.4, may affect the stability and absorption of the active ingredients [5, 11]. Intestinal activity, including motility and absorption, can also influence the amount of medication that is delivered [68]. Finally, hepatic conditions, including interactions with hepatic enzymes and the liver’s defensive cells, can metabolize or eliminate certain active ingredients [7, 69]. Once oral medications overcome these exogenous barriers, they must cross the systemic physiological barriers of the organism to reach the kidney. These barriers include the immune system, which may recognize and eliminate medications as if they were foreign substances, and the microenvironment of the target organ, in this case, the kidney (Figure 3). In this regard, systemic barriers can influence the distribution, metabolism, and elimination of medications, which can affect their efficacy. Therefore, for therapies aimed at treating kidney diseases to be effective, they must overcome both exogenous and systemic barriers and reach the target organ in an active and effective form.

### 4.1. Nanoparticles as a Novel System to Overcome External (Exogenous) Physiological Barriers

Although conventional pharmaceutical formulations such as tablets, capsules, and syrups may be compromised by various exogenous pathway factors, including pH variations, digestive enzymes, and other gastrointestinal conditions, numerous studies have demonstrated that formulations incorporating inorganic and organic nanoparticles effectively circumvent these biological barriers owing to their distinct physicochemical attributes [70]. Furthermore, it has been reported that nanoparticles with diameters ≥ 300 nm predominantly accumulate in the hepatic tissue, while those exceeding 500 nm tend to be sequestered within the gastrointestinal mucus layer [12, 71]. Upon hepatic deposition, nanoparticles encounter Kupffer cells, the liver‐resident macrophages integral to immune surveillance. These phagocytes exhibit preferential uptake of larger nanoparticles exhibiting hydrophobic surface chemistries, thereby constituting a major clearance mechanism [72, 73]. Inorganic nanoparticles such as those composed of iron oxide, zinc oxide, titanium dioxide, gold, silver, and silicon establish multifaceted interactions with Kupffer cells, the liver‐resident macrophages responsible for nanoparticle recognition and clearance. These interactions are governed by a combination of physicochemical surface properties, primarily hydrophobicity/hydrophilicity and surface charge of nanoparticles. The degree to which Kupffer cells recognize and internalize these nanoparticles depends on the subtle balance and interplay between these surface characteristics, making their bio‐nano interface highly dynamic and context‐dependent [74]. Notably, positively charged nanoparticles with sizes ≥ 10 nm, particularly those based on silver, gold, and silicon, have been engineered to evade Kupffer cell‐mediated clearance, enhancing renal targeting capabilities by facilitating systemic circulation and renal accumulation [75].

### 4.2. Nanoparticles as a Novel System to Overcome Internal (Endogenous) Physiological Barriers

Minerals and natural materials have been used since ancient times to treat injuries and other physiological conditions in humans. As a result, modern research has focused on metallic biomaterials and the synthesis of nanostructures with significant potential for therapeutic action [12]. Currently, nanometric drug engineering allows for the modification of properties such as size, shape, charge, porosity, surface, and hydrophobicity of nanoparticles. This is very promising for the development of drugs capable of overcoming the barriers constituted by the human immune response to reach the pharmacological target. This is achieved through careful selection of materials and immune antagonists, as well as manipulation of the immune response to nanoparticles, which requires a deep understanding of the immune system and the properties of nanoparticles for the development of effective and safe therapies [76].

As mentioned in Figure 3, the human body has a complete arsenal of defenses that are activated in the presence of foreign particles in the organism, with the aim of neutralizing and eliminating them [12, 77]. According to Mariani et al., the endogenous defense systems of the organism are initially composed of cellular defenders: (1) myeloid cells such as macrophages and neutrophils and (2) lymphoid cells such as T‐lymphocytes and B‐lymphocytes, which are responsible for identifying and neutralizing invaders through phagocytosis. In this regard, the use of polymeric biomaterials and their interaction with the immune system has been widely investigated, showing that their biocompatibility and immunogenicity largely depend on their chemical composition and structure. Some polymers can induce beneficial anti‐inflammatory responses, while others may trigger adverse effects such as necrosis or functional changes in the tissue, depending on the context and the specific type of polymer used [78].

Immunomodulation is an important factor to consider for curing or counteracting renal conditions, with nanotechnology playing an important part. Several authors report that nanoparticles sized between 40 and 100 nm provoke strong proinflammatory responses, while nanoparticles below 40 nm may go unnoticed by the immune system [6, 71]. Furthermore, it has also been reported that the shape and surface of nanoparticles (i.e., electrostatic charge and hydrophobicity) can either increase or decrease nanoparticle uptake by the immune system [6]. On the other hand, it has been reported that metallic biomaterials can have favorable interactions with the human immune system, making them suitable biomaterials to overcome the endogenous limitations of the organism [79].

In the immune system of organisms, immunoglobulins recognize nanoparticles or other foreign particles [12]. Typically, IgG or IgM are responsible for activating the classical pathway after binding to foreign material [12, 80, 81]. B cells originate with IgM and IgD receptors until they undergo isotype switching, allowing them to express IgG, IgE, or IgA [12, 82]. Regarding the uptake and elimination of nanoparticles, it has been reported that immunoglobulins easily capture highly anionic or cationic nanoparticles, thereby inducing their phagocytosis. It has also been reported that hydrophobic nanoparticles easily activate the innate immune system.

### 4.3. Microenvironment of the Kidney

It is challenging for many nanoparticles to reach the kidney without being neutralized by the body’s defense system. However, it has been historically and scientifically established that some inorganic compounds exhibit a certain degree of tolerance in the human body and are used in the diagnosis and treatment of various diseases [83]. The biocompatibility of inorganic nanoparticles can vary considerably depending on their composition and design, which grants certain inorganic nanoparticle systems strategic advantages in counteracting specific human ailments [5, 84, 85]. Additionally, the physicochemical characteristics of inorganic nanoparticles, such as their size, which can reach down to 5 nm, their modifiable electrostatic charge, and the possibility of combining them with bioactive and stabilizing compounds from medicinal plants (i.e., flavonoids, terpenes, polyphenols, etc.), enhance their credibility when selecting pharmaceutical forms to protect the kidney from potential damage caused by free radicals [13, 86, 87].

**Figure 4:**
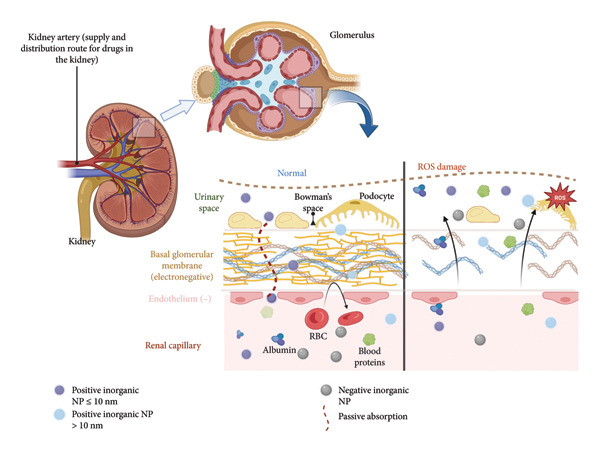
Glomerular structure of a healthy kidney and a kidney damaged by oxidative stress. In a damaged kidney, the glomerular fenestrations are affected by ROS, altering their physiological function. On the other hand, in a healthy kidney, the natural filtration system prevents the passage of blood components and particles larger than 10 nm. Therefore, when designing nanoparticles for overcoming the barriers of the renal microenvironment, the size and electrostatic charge need to be considered.

The glomerulus is a specialized capillary network that facilitates the exchange of substances between blood and urine [88]. Understanding the complexity of the renal filtration system is crucial for developing new therapies for kidney diseases. Nanotechnology holds great promise in this field, enabling the creation of nanoparticles designed to specifically interact with kidney cells and membranes. Once in the kidneys, metallic nanoparticles must surpass the renal glomerular filtration system to reach the urinary space (Figure 4). As illustrated, free radicals are capable of irreversibly damaging the glomerular system. Therefore, developing drugs that can protect the kidney against damage from ROS is paramount to ensuring health. Under normal circumstances, glomerular fenestration constitutes a physical barrier for blood filtration, effectively limiting the passage of particles and components of the blood. However, due to the size of Bowman’s fenestration, nanoparticles with a particle size of ≤ 10 nm can successfully traverse the glomerular filtration barrier. Furthermore, due to the antioxidant potential of certain inorganic materials and their respective stabilizers, such as those derived from medicinal plants, the use of inorganic nanoparticles as a preventive measure against the effects of ROS on the kidney is supported.

## 5. Synthesis of Inorganic Nanoparticles

**Figure 5:**
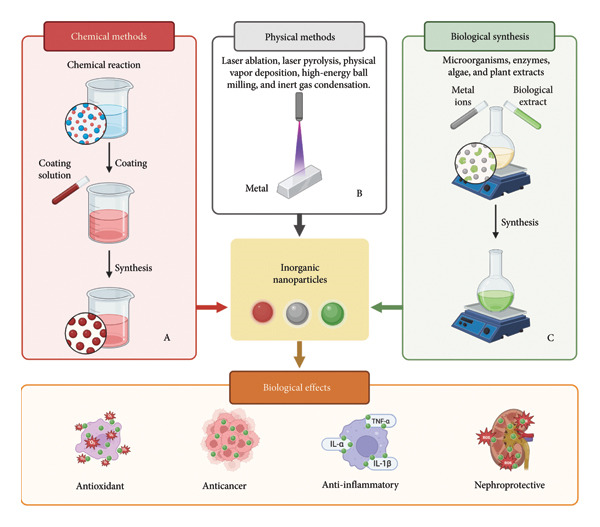
Strategies for the synthesis of inorganic nanoparticles. (A) Chemical approaches: hydrothermal and solvothermal synthesis, polyol synthesis, Brust–Schiffrin and Turkevich methods, sol–gel processing, and chemical vapor deposition. (B) Physical approaches: laser ablation, laser pyrolysis, physical vapor deposition, high‐energy ball milling, and inert gas condensation. (C) Biological approaches: green synthesis via microorganisms, enzymes, and plant‐derived extracts.

Inorganic nanoparticles can be produced by breaking down bulk materials (i.e., the top‐down approach) or by integration of individual atoms and molecules (i.e., the bottom‐up approach). The bottom‐up approach largely pertains to chemical methods of preparation of nanoparticles, whereas the top‐down approach is associated with many mechanical/physical methods (Figure 5). Biological synthesis of nanoparticles involves the use of organisms such as microorganisms or plant extracts. The natural compounds used function as reducing agents, capping agents, and/or stabilizing agents and can have bioactive properties important for treating various diseases. This section highlights the different methods used for synthesizing inorganic nanoparticles, with bioactive phytochemicals being incorporated with nanoparticles during synthesis or after the formation of nanoparticles (e.g., through conjugation or noncovalent interactions). Phytochemicals can be loaded on metal nanoparticles produced using physical and chemical methods by adsorption [89].

### 5.1. Physical Methods

Physical methods often employ a top‐down approach that is solvent‐free and environment friendly. Physical methods include laser ablation (LA), laser pyrolysis (LP), physical vapor deposition (PVD), high‐energy ball milling (HEBM), and inert gas condensation (IGC). In the LA technique, surface atoms from a solid target material are vaporized using a focused high‐intensity laser beam [90]. This results in the formation of a plasma plume that eventually cools, condensing ablated species into nanoparticles that nucleate and grow. The yield and size of laser‐ablated nanoparticles in a particular solvent heavily depend on the laser parameters, such as the laser beam wavelength, fluence, repetition rate, and duration of the laser pulse, as well as the ability of the target to absorb light and the properties of the liquid [91]. This process can occur in a vacuum chamber, with the technique known as pulsed laser deposition [92]. The laser energy vaporizes the target material, creating a plasma plume that expands toward a substrate. The plasma plume consists of ions, molecules, and/or aggregates. The energetic species deposit and form a thin film. Electrically charged particles are formed, with the nucleation process significantly influenced by electron and ion species. PVD involves vaporizing solid precursors using physical methods (e.g., evaporation, sputtering) followed by their condensation onto a substrate to form nanoparticles [93]. The solid material (i.e., bulk material, liquid source) is vaporized into particles that are transported through a vacuum or low‐pressure environment to the substrate. Upon reaching the substrate, the particles condense and form a thin film or nanostructures. Thickness and morphology of deposits depend on factors such as rate of supply of atoms to the region of deposition, rate of removal of energy from saturated atoms, and rate of removal of recent nucleation [94]. LP is a vapor‐phase synthesis technique that uses a focused laser beam to heat reactant vapors/gases, inducing decomposition and formation of nanoparticles [95]. The final nanoparticle forms through initial particle formation followed by coalescence of smaller particles. The resulting nanoparticles are collected as a dry powder, often in a collection chamber. HEBM is a mechanical deformation process, containing a low number of balls that function as the grinding medium inside the container [96]. They can move freely and have adequate space to gain high momentum before any impact. A motor that shakes the container in a planetary motion provides the system with high energy levels capable of milling even the most challenging materials. Parameters such as milling speed, time, and ball size affect the physical and morphological properties of the nanoparticles produced [97]. IGC is a versatile method for synthesizing nanoparticles, starting with vaporizing a material in a high‐vacuum environment [98]. A cold, inert gas is introduced into the chamber for colliding with the evaporated atoms. This results in the evaporated atoms losing kinetic energy and the condensation into clusters or nanoparticles. The clusters of nanoparticles are collected on a substrate or as a powder. The size of the prepared nanoparticles can be regulated based on the chamber pressure, inert gas injected into the chamber, and temperature [99]. In general, physical methods have advantages such as time efficiency and the absence of toxic compounds. However, common drawbacks include excessive energy consumption, high cost, difficulties in the production of nanoparticles on a large scale, and polydispersity of nanoparticles.

### 5.2. Chemical Methods

Chemical synthesis methods include hydrothermal synthesis, polyol synthesis, the Brust–Schiffrin method, the Turkevich method, solvothermal synthesis, the sol–gel method, chemical vapor deposition (CVD), and plasma‐enhanced CVD synthesis (PECVD). Many synthesis techniques can involve the use of microwaves for heating, as it is energy efficient and reduces reaction times for high‐temperature calcination, enabling fast and reproducible results. Inorganic and organic reducing agents are used to reduce ions, resulting in metal formation, agglomeration into oligomeric clusters, and creation of metallic colloidal particles [100]. Agglomeration is prevented using protective agents (e.g., polyethylene glycol [PEG], polymethylmethacrylate, pyrrolidone, and poly(methacrylic acid)) that adsorb or bind on the surface of nanoparticles [101]. Typical CVD consists of two steps: (1) transportation of gas‐phase materials into the chamber and gas‐phase reaction and (2) the formation/deposition of the final nanomaterial on the substrate [102]. The precursors exist in three different states (i.e., solid, gas, and liquid), produced as vapors in the reactor under conditions required for nucleation of particles. Chemical reactions occur at extremely high temperatures in order to deposit solid films from the vapor phase. How well nanomaterials are deposited depends on the precursor content, reaction rate, and temperature. PECVD is a CVD process where plasma (e.g., from microwaves or radiofrequency fields) is used to excite precursor gases, allowing for deposition on substrates at lower temperatures. The hydrothermal method can yield monodisperse particles with high crystallinity. The synthesis of nanoparticles involves reacting chemical solutions of precursors in a sealed, heated chamber, promoting nucleation and crystal growth at temperatures typically above the boiling point of water and above ambient pressure [103]. A closed vessel or hydrothermal reactor is required for creating high‐temperature and ‐pressure conditions for dissolving and recrystallizing materials in water. The composition, surface chemistry, particle size, and morphology can be regulated by adjusting the reaction time, temperature, pressure, solvent properties, additives, and solution composition. Solvothermal synthesis is a very similar technique, with the key difference being the use of solvents other than water. Metal oxides can be created using the sol–gel technique from a solution of chemicals that acts as a precursor to a combined polymer network or discrete particles [104]. The sol–gel process begins with selecting suitable precursors (e.g., metal alkoxides, salts) for initiating hydrolysis and condensation reactions and controlling reaction conditions. These compounds dissolve in solvents such as alcohol or water to create a homogeneous solution. Hydrolysis occurs when the precursors react with water, for example, when alkoxide groups are replaced with hydroxyl groups. This is followed by condensation, where hydroxyl groups from adjacent molecules interact to form metal–oxygen–metal bonds, creating a three‐dimensional network. These reactions lead to the formation of colloidal particles that gradually develop into a gel. During drying, the solvent is removed from the gel, producing a porous solid (i.e., xerogel), and aging allows further condensation and structural reorganization, improving mechanical strength and stability. Heat treatment, particularly calcination, is used to convert the xerogel into crystalline nanoparticles with specific properties. Reaction conditions influence sol stability and nanoparticle properties, while temperature and solvent polarity affect solubility and stability of precursors and intermediates as well as hydrolysis and condensation rates [105]. Reaction time, precursor concentration, and temperature also affect gelation behavior. Polyol synthesis is used to produce noble metal and magnetic particles [106]. It involves suspending the metal precursor in a solvent made of glycol, heating the solution to a refluxing temperature, and controlling the reduction and growth of nanoparticles through kinetic control of nucleation and growth. The heated polyol solution functions to dissolve the metal precursor and as a reducing agent for the reduction of metal. Other reducing agents can be used along with surfactants for controlling the shape and size of nanoparticles produced using thermal decomposition [107]. The Brust–Schiffrin method is a two‐phase synthesis technique used to produce thiol‐protected metal nanoparticles (i.e., usually in a toluene‐water system) [108, 109]. Typically this method consists of three steps. Step 1 involves phase transfer of metal ions from an aqueous to an organic phase (e.g., toluene, benzene) with a phase transfer reagent (e.g., tetraoctylammonium bromide). In Step 2, organochalcogen‐containing ligands (usually RSH) are added to the separated organic phase, during which metal cations are reduced (e.g., Au^3+^ to Au^+^). For the last step, metal ions residing in the separated organic phase are reduced into M^0^ by a reducing reagent like NaBH_4_, during which organochalcogenolate‐protected metal NPs are formed. The type and concentration of reactants, reaction conditions (e.g., temperature, time, pH), and the stabilizing agents used all impact the size, shape, and stability of nanoparticles produced. The Turkevich synthesis, or the seed‐mediated growth mechanism, involves four steps [110]. The first step is a partial reduction of the metal precursor and the formation of small clusters from the metal monomers. In a second step, these clusters form seed particles while the remaining metal ions are attracted and attached in the electronic double layer of the seed particles as co‐ions. The third and fourth steps comprise the reduction of the ionic metal (i.e., first slowly, then fast), whereby the generated metal monomers grow exclusively on top of the seed particles’ surfaces until the precursor is fully consumed. Synthesis parameters such as concentration, pH value, order of reactant addition, and synthesis temperature need to be carefully considered, as they influence the size of nanoparticles produced. In general, chemical methods for synthesis of nanoparticles offer advantages such as high versatility, cost‐effectiveness, and precise control over size and shape. The main disadvantage is the potential toxicity of the nanoparticles produced due to the involvement of chemicals during synthesis.

### 5.3. Biological Synthesis

Nanoparticles can be synthesized by means of biological entities both intracellularly and extracellularly [111]. Together with chemical methods, biosynthesis techniques follow a bottom‐up approach involving oxidation/reduction reactions. To create metal and metal‐oxide nanoparticles, biological synthesis methods use biological systems such as fungi, yeast, algae, bacteria, and different plant‐based extracts. The biological agent or extract is mixed with the metal ion solution, with its metabolites converting the metal ions into their elemental form. The reduced metal ions aggregate to form nanoparticles, which are then stabilized by the biological molecules present in the solution. Microorganisms are attractive for the synthesis of nanoparticles due to the ability to easily cultivate them, their fast growth rate, and their ability to grow at ambient pH, temperature, and pressure conditions [112]. Microbial synthesis of nanoparticles can be categorized into either biosorption or bioreduction. Biosorption involves the binding of metal cations present in aqueous media to the cell wall of the organism. Possible mechanisms for biosorption of metals onto microbial species include physisorption, precipitation, ion exchange, and complexation, leading to the formation of stable nanoparticles. Microbes secrete polysaccharide substances, which mostly possess anionic functional groups having the potential to attract cations from aqueous solutions. Cell wall components like peptidoglycan in bacteria, chitin and β‐1,3‐glucan in fungi, and pectin in plants can be used as agents to reduce metal ions for the synthesis of nanoparticles [113]. In bioreduction, biomolecules such as enzymes (e.g., reductases) from cells are used as reducing agents. Plant extracts, which contain high levels of phytochemicals, including flavonoids and phenolic acids, can also effectively function as reducing and capping agents [114]. The plant material is first heated in a suitable solvent (e.g., water, ethanol) to extract the phytochemicals and then filtered and purified before combining with a solution containing metal salts [115]. The extracts and the precursor salt coordination complexes are formed between the metal ion and the phytochemicals of the extract before the nanoparticles grow through nucleation and adsorption. The final shape of the nanoparticles occurs during termination. Plant‐based synthesis of nanoparticles offers several advantages over microbe‐based methods, like ease of access to material, lower time consumption, and simpler synthesis processes due to the elimination of cell culture maintenance and potentially lower costs [116]. The amount of phytochemicals on nanoparticles from synthesis is usually sufficient for providing the desired therapeutic response. In general, green synthesis methods use molecules that are significantly less toxic to the environment than those used in traditional methods. Nanoparticles produced using plant extracts have improved compatibility with living organisms and decreased adverse effects. Having said this, there are a few challenges associated with many biological methods, such as lack of shape and size control, low production quantity and rate, and process scale‐up.

## 6. Inorganic Nanoparticles With Nephroprotective Potential

Inorganic nanoparticles hold significant promise for various aspects of kidney therapy, including drug delivery, targeted therapy, and diagnostics. They can be designed to target specific kidney tissues and for delivering drugs effectively. In particular, nanoparticles made of gold, silver, metal oxides, and selenium have been extensively studied for their nephroprotective potential. They can be used to reduce inflammation and oxidative stress and for promoting cell repair, making them potentially valuable in treating kidney diseases. Inorganic nanoparticles with magnetic properties, such as iron oxide nanoparticles (IONPs), are commonly used for magnetic hyperthermia and magnetic resonance imaging (MRI), while inorganic nanoparticles with high extinction coefficients from light absorption, such as gold nanoparticles (AuNPs), are excellent agents for photothermal therapy (PTT). Densely packed inorganic metal nanoparticles can selectively scatter and/or absorb high‐energy gamma/x‐ray radiation and can serve as radioenhancers for radiotherapy. Although commonly used for cancer diagnosis and therapy, these strategies allow for better targeting of cellular components within target tissues, allowing for more localized treatment [117]. Despite the efficacy of inorganic nanoparticles, concerns about long‐term biocompatibility and toxicity remain. In this context, plant‐derived antioxidants represent a complementary and promising strategy. These natural compounds, such as polyphenols, flavonoids, and terpenoids, not only possess potent antioxidant and anti‐inflammatory properties but also exhibit high biocompatibility and low toxicity. Moreover, they can be combined with nanoparticle systems to generate synergistic effects, enhancing therapeutic efficacy while reducing adverse effects [118–120].

### 6.1. AuNPs

AuNPs have medicinal potential in nephroprotection, with their efficacy and safety dependent on their physical factors (e.g., size, shape, surface charge) and dose [121–123]. Smaller AuNPs (i.e., smaller than 10 nm) have low nephrotoxicity and accumulation in the kidneys, as they can pass the glomerular filtration barrier, while larger AuNPs (i.e., between 20 and 100 nm) are sequestered in the kidney’s mesangium and may enhance kidney toxicity. Particles larger than 100 nm are primarily retained by the liver and spleen. Spherical AuNPs are generally easier to manufacture, and their biological interactions are more predictable due to the significant amount of studies carried out, compared to rod‐ or star‐shaped particles that have variable biodistribution patterns and clearance rates [124]. Neutral or zwitterionic charged AuNPs circulate longer and are less harmful compared to positively charged AuNPs [125, 126]. The kidneys can be protected against oxidative stress‐induced tissue damage and malfunction due to the antioxidant and anti‐inflammatory effects of AuNPs [124, 127]. Polymer coatings (e.g., using PEG, chitosan, or dextran) improve the biocompatibility, stability, and circulation time of AuNPs by reducing immune reactions and renal accumulation [128]. They also enable functionalization of AuNPs for targeted and combination therapies. Stability is especially important, as aggregation can modify the size and distribution of AuNPs in organs, leading to unexpected toxicities. The nephroprotective activities are from the surface‐decorated agents (e.g., peptides, proteins, polysaccharides, and plant extracts) that are incorporated on the AuNPs from their synthesis. Cells are protected from oxidative damage by the neutralizing ability of surface agents against ROS and reactive nitrogen species. The functional groups of AuNPs enable them to imitate naturally occurring antioxidant enzymes (e.g., superoxide dismutase [SOD] and catalase [CAT]), boosting catalytic activity. The AuNPs regulate accumulation of antioxidant enzymes SOD, CAT, and glutathione peroxidase (GPx) and inhibit superoxide radicals at the injury site for causing nephroprotection [129–132]. An interaction between the unpaired electrons from the free radical and the conduction band electrons of AuNPs enhances the antioxidant and anticancer properties [133]. AuNPs are used for shielding renal tissues from being damaged by apoptosis by protecting mitochondrial integrity, impeding fibrosis, and altering apoptotic pathways [134–136]. AuNPs may help preserve renal function and reduce tissue injury by modulating inflammatory signaling pathways and reducing the production of proinflammatory cytokines [137, 138]. The nephroprotective function of AuNPs in AKI, a tubule‐interstitial injury without remarkable glomerular function abnormalities, is well studied [139, 140]. When kidneys are damaged, the proximal tubules are commonly affected, with cellular necrosis occurring due to either direct harmful effects on the vascular wall or inadequate oxygen supply resulting from damage to the capillary wall [141, 142]. The use of nephrotoxic drugs and their byproducts results in the buildup of altered cellular material in the tubule [143]. The use of AuNPs for sub‐AKI can result in mitigation in the inflammatory profile (i.e., inducing anti‐inflammatory T helper 2 response) and tubular damage indicators (i.e., reduction in interstitial damage) without glomerular changes and nephrotoxicity at a low dosage (i.e., 10 μg/kg/day). In addition, AuNPs can be used to lessen renal damage caused by chemicals (e.g., cadmium). For example, when administered at 5 mg/kg, AuNPs (i.e., 25–30 nm) synthesized using aqueous leaf extracts (e.g., *Nymphaea lotus*, NL) had ameliorative effects as seen by changes in levels of kidney function markers (e.g., serum urea and creatinine) and inflammatory markers (e.g., interleukin‐6 [IL‐6], nuclear factor‐κB [NF‐κB]) [144]. Usually, restoration of normal kidney function is associated with a reduction in levels of serum urea and creatinine [145]. Improvements in glomerular tufts and partial regeneration of renal tubules indicate a protective effect. At higher doses, renal tubules appear damaged with an increase in urinary spaces, indicating possible toxicity. The NL‐AuNPs can counteract the increased oxidative stress caused by exposure to high amounts of cadmium by reducing lipid peroxidation and tissue damage. The mode of action comprises a combination of antioxidant, anti‐inflammatory, and cellular protective activities that preserve renal function and integrity in the presence of toxicity [146]. AuNPs can be used for treatment of kidney diseases (e.g., diabetes). For example, administration of 10 mg/kg AuNPs (40–45 nm), synthesized using *Fritillaria cirrhosa* plant extract, declined aspartate aminotransferase (AST), alanine aminotransferase (ALT), alkaline phosphatase (ALP), blood glucose, and glycosylated hemoglobin levels and increased serum protein content to the normal range, compared to the diabetic group (i.e., without treatment with AuNPs) [147]. AuNPs produced using jujube aqueous extract (i.e., *Ziziphus jujuba*) at a dose of 0.5 and 1 mg/kg reduced malondialdehyde (MDA) and amounts of lipids and lipoproteins (e.g., triglycerides, total cholesterol, high‐density lipoproteins, and low‐density lipoproteins) in the bodies of the diabetic group [148].

### 6.2. AgNPs

AgNPs can be used to treat kidney damage caused by chemicals, medications, or illnesses. This is possible due to their free radical scavenging capability and ability to reduce oxidative stress‐induced damage in renal cells [149]. AgNPs can be modified with ligands or polymers on their surface to target specific infectious or malignant tissues, for improving therapy and for decreasing the toxicity in healthy tissues [150]. Drugs can be loaded on nanoparticles, making it possible to achieve synergistic effects for enhancing treatment of various diseases such as renal carcinoma [151]. Compared to the diabetic untreated group, the kidney weight, kidney volume, and length of kidney structures decreased significantly after treatment of the group with diabetic nephropathy using 10 and 40 μg/kg of AgNPs (i.e., made using *Pistacia atlantica* aqueous extract) [86]. The levels of blood glucose and urea were decreased significantly in the diabetic groups treated with AgNPs. AgNPs (i.e., ∼50 nm, 20 and 30 mg/kg) produced using *Carissa carandas* aqueous plant extract can be used for nephroprotection, for ameliorating oxidative stress and carcinogenesis, with reduced levels of serum marker molecules (i.e., uric acid, blood urea nitrogen [BUN], and creatinine) and tumor marker enzymes (i.e., gamma glutamyl transpeptidase [GGT], xanthine oxidase, and lactate dehydrogenase [LDH]), compared to the group treated with the toxic carcinogen diethylnitrosamine but without treatment with AgNPs [152]. Proinflammatory cytokines and inflammatory mediators such as tumor necrosis factor alpha (TNF‐α), IL‐6, and NF‐κB were downregulated, and endogenous antioxidant enzymes such as SOD, CAT, GPx, and glutathione S transferase were significantly upregulated. The boost in GSH synthesis from the use of AgNPs counters the favorable effects of antioxidant metabolic activity [153]. The reduction in oxidative stress triggers the activation of nuclear factor erythroid 2‐related factor 2 (Nrf2), a transcription factor that regulates the expression of antioxidant and detoxification genes. Nrf2 binds to antioxidant response elements (AREs) in the promoters of genes involved in GSH synthesis, such as glutamate–cysteine ligase (GCL), which catalyzes the rate‐limiting step in GSH production. By enhancing the activity of Nrf2, AgNPs upregulate GCL expression, leading to increased intracellular levels of GSH [154]. High levels of GSH help to eliminate N‐acetyl‐*p*‐benzoquinone imine (NAPQI), a reactive metabolite that can cause cellular damage [155]. The reduction in oxidative stress and inflammation helps to prevent tubular cell necrosis, renal tubular vacuolation, and structural damage (e.g., induced by nephrotoxic agents), improving overall kidney function and maintaining renal tissue integrity. Levels of tumor promotion enzymes such as thymidine phosphorylase and ornithine decarboxylase decrease after treatment with AgNPs. Histological studies reveal tissue repair, demonstrating the therapeutic potential of AgNPs against renal injury and disease [156]. Treatment with AgNPs (20–70 nm), which were synthesized using Arabic gum, significantly attenuated ROS generation caused by nephrotoxic agents, having a protective effect on AKI [157]. Administration of 15, 30, 60, and 120 μg/kg AgNPs significantly decreased kidney injury molecule‐1 and neutrophil gelatinase‐associated lipocalin expression. However, these biomarkers for kidney injury and disease were expressed at very high levels in the untreated group (i.e., AKI without treatment with AgNPs) [158]. In another study, AgNPs (∼30 nm average size, 10 mg/kg) synthesized using *Bryophyllum pinnatum* leaf extract were used to treat urolithiasis (i.e., kidney stones) with a substantial increase in serum total protein, albumin, and globulin and a significant decrease in AST, ALT, creatinine, BUN, calcium, and phosphorus, compared to the untreated group (i.e., with urolithiasis but not treated with AgNPs) [159]. AgNPs show potential to prevent kidney damage as enzymatic function is restored and urea and creatinine levels are downregulated after administration of AgNPs. Mild degenerative changes and restoration or maintenance of kidney parenchyma were observed in treated groups when compared to the untreated group. Treatment of nephrotoxicity with AgNPs at 50 μg/kg dose led to significant improvements in proximal and distal convoluted tubules. Better results were observed at doses of 100 and 150 μg/kg for AgNPs, with improved structure of glomeruli, well‐organized tubules and glomeruli, and normal nuclear organization in the epithelium of collecting tubules [160]. Clear and wide lumens were observed in the renal tubules. AgNPs can have other important biomedical applications, as the Ag^+^ ions released from AgNPs can block bacterial replication, disrupt numerous metabolic pathways, and destroy cancer cells [161].

### 6.3. Metal Oxide Nanoparticles (MONPs)

MONPs such as CuO, ZnO, IO, and TiO_2_ NPs are biodegradable, biocompatible, cost‐effective, and environmentally safe, especially those produced using biological materials (i.e., green synthesis) [162, 163]. MONPs can be biodegraded through several mechanisms, including dissolution, oxidative degradation, and enzymatic degradation [164, 165]. The specific mechanisms depend on the composition, size, and surface properties of MONPs as well as the surrounding environment. MONPs have antioxidant, antimicrobial, anti‐inflammatory, and anticancer properties for treating a variety of diseases [166–169]. Zinc oxide (ZnONPs, 30–40 nm) produced using leaf extract of *C. sinensis* has ameliorative potential against nephrotoxicity [170]. ZnONPs at a dose of 25 ppb reversed damage caused by nephrotoxic chemicals and was involved in reducing the levels of ALT, AST, and creatinine and increasing albumin and total protein levels. Biochemical analysis showed improvement in kidney tissues. ZnONPs prevent decreases in renal SOD, CAT, and glutathione reductase and increase in renal MDA levels. The proportion of viable kidney cells is significantly elevated, and the proportion of apoptotic and necrotic kidney cells is significantly reduced, compared to the group exposed to the nephrotoxic agent only [171]. The level of renal transforming growth factor beta 1 (i.e., TGF‐β1, inflammatory cytokine) decreased in the group pretreated with ZnONPs. The nuclear factor‐E2‐related factor, heme oxygenase‐1, and endothelial nitric oxide synthase expression genes were upregulated, while Bcl‐2‐associated X protein expression was downregulated in kidney tissue via ZnONPs. The blood glucose level, renal oxidative stress markers, and glomerular basement membrane thickness can be reduced with ZnONPs (< 100 nm, 2.5 mg/kg) for improving diabetic nephropathy and enhancing renal function [172]. In addition, expression of inflammatory cytokines (i.e., TGF‐β1, TNF‐α) and abnormal angiogenesis were inhibited, and the development of podocyte injury was delayed. Anemia associated with end‐stage renal disease and CKD can be treated using IONPs (60–70 nm), synthesized using *Petroselinum crispum* leaf extract [173]. Levels for hemoglobin, red blood cells, mean corpuscular volume, mean corpuscular hemoglobin, mean corpuscular hemoglobin concentration, and packed cell volume increased to normal in anemic rats treated with 27 ppm IONPs. Treatment of anemia with IONPs improved the pathological alterations in the kidneys to variable degrees. Microscopic examination of the kidneys of anemic rats treated with IONPs revealed distinct improvements in renal tissue histology compared to the control group (i.e., with anemia). Renal blood vessels and glomerular blood capillaries were less congested with mild vacuolation of the endothelial cell lining of the glomerular tuft. Degenerative changes in the epithelial cell lining of the renal tubules in the form of cloudy swelling were also mild. Due to their intrinsic chemical properties, IONPs can be used for MRI, allowing the distinction between renal intrinsic disease and obstructive uropathy, as well as to identify the signs of kidney rejection [174]. The surface of these particles can also be conjugated with ligands (e.g., antibodies) for targeting kidney vasculature and inflamed endothelial cells [175, 176]. This enables assessment of the proinflammatory mediator vascular cell adhesion molecule‐1 expression associated with ischemia‐reperfusion injury. The use of green synthesized cerium oxide nanoparticles (CeONPs), using *Mentha royleana* leaves extract, led to significant inhibition of alpha‐glucosidase, sucrase, and alpha‐amylase for treating hyperglycemia and effectively decreased ROS levels [177]. The response of these CeONPs was dose‐dependent, with 500 and 1000 μg/mL being proven to be effective doses. The administration of cerium oxide (ceria) nanoclusters (NCs, 1 mg/kg), especially those modified with PEG (i.e., PEG600, PEG2000), effectively treated CKD [178]. In addition to acting as exogenous ROS scavengers, the ceria nanoclusters can activate endogenous ROS scavengers by upregulating Nrf2 and downregulating Keap1 in the kidneys. The downstream antioxidant enzymes, including HO‐1 and SOD, were highly expressed. The NCs could significantly inhibit the macrophage infiltration, with IL‐1β and IL‐6 being effectively inhibited. The expression of nuclear factor‐κB was significantly reduced with fewer p65 proteins activated in treated kidneys. These results indicated that ceria NCs could effectively inhibit oxidative stress and inflammation associated with CKD. The antifibrosis activity of NCs was further verified by the detection of fibrogenic cytokines, COL‐1 and TGF‐β1, which were all significantly suppressed by NCs. The mRNA expression of caspase‐3 and the protein expression of Bax were decreased, while the protein expression of Bcl‐2 was increased in kidneys with CKD treated with NCs. The apoptotic indexes of kidney cells were much lower from treatment with NCs compared to the untreated group (i.e., with CKD). Ligands and additional therapeutic molecules can be loaded for CeONPs to improve kidney targeting and function (e.g., from AKI) [179, 180]. Specific types of MONPs, including those synthesized using extracts from plants, have been shown to generate ROS in cancer cells, including those of kidneys, as part of their anticancer mechanism, leading to mitochondrial, DNA, and protein damage [181–183].

### 6.4. Selenium Nanoparticles

Selenium (Se) is a highly selective and biocompatible element and an important antioxidant used in biological activities such as growth, metabolism, and hormonal homeostasis [184]. It is involved in the metabolism of hydrogen peroxide and lipid hydroperoxides, which protect cells from the noxious effects of free radicals formed during oxidation processes. The risk of acute/chronic renal failure or even death can increase when there is a lack of selenium in the body. The efficacy of selenium for therapy is significantly improved when it is used in nanoformulations [185]. SeNPs can regulate innate immunity to intervene in disease development and boost anticancer, anti‐infection, and anti‐inflammation treatments [186]. These NPs are cytotoxic toward cancer cells by activating and recovering different T cells for adaptive immunity regulations. Due to antioxidant and anti‐inflammatory activities, SeNPs have nephroprotective properties, preventing apoptosis of renal cells undergoing oxidative stress (e.g., from toxic agents) and preserving their structural integrity and functionality [187]. SeNPs (∼50 nm) synthesized using aqueous leaf extract of the plant *Spermacoce hispida* (Sh‐SeNPs) can be conjugated with the biologically active ligand s‐allyl glutathione (SAG) to produce SAG‐Sh‐SeNPs to enhance the biological activity in the kidneys [188]. Pretreatment against toxicity‐induced kidney damage with either Sh‐SeNPs or SAG‐Sh‐SeNPs alone (i.e., a dosage of 0.2 mg/kg/day for both) eased the pathological alteration of biochemical parameters (i.e., AST, ALT, ALP, GGT, LDH, bilirubin, triglycerides, urea, uric acid, creatinine, and cholesterol in serum; albumin and total protein in plasma). Due to the synergistic effect, SAG‐Sh‐SeNPs showed an enhanced protective effect against nephrotoxicity in comparison to Sh‐SeNPs treatment alone. Pretreatment with these NPs (i.e., before administration of the toxic agent) protected the kidneys against severe tubular and glomerular damage [189]. This prevented oxidative stress and preserved the level of antioxidants against toxicity in the kidneys. The concentrations for ROS, nitric oxide (NO), and MDA were reduced and much closer to normal levels compared to the group treated with the nephrotoxic chemical only [190–192]. A decrease in NO levels reduces the amount of peroxynitrite radical generated by ROS. Peroxynitrite mediates formation of 3‐nitrotyrosine‐protein adduct and results in ubiquitination of the nitrated protein [193, 194]. This leads to protein, DNA, and lipid damage, which causes necrosis. SAG‐Sh‐SeNPs prevented the depletion of GSH and vitamin C content and increased enzymatic activity, as seen by the increase in levels of SOD, CAT, and GPx. Pretreatment with Sh‐SeNPs and SAG‐Sh‐SeNPs preserved the mitochondrial oxidative phosphorylation (OXPHOS) function. SAG‐Sh‐SeNPs showed a higher protective effect on mitochondrial function in comparison to Sh‐SeNPs. Mitochondrial complex I (NADH‐dehydrogenase) and III (cytochrome‐c‐reductase) activities were very similar to normal levels after pretreatment with these NPs. This is in contrast to results from the group with nephrotoxicity (i.e., without treatment with SeNPs), indicating nitration of various proteins such as aldehyde dehydrogenase, citrate synthase, and ATP synthase in mitochondria [195]. Mitochondrial CAT, SOD, and GPx are nitrated, resulting in depletion of mitochondrial oxidants. NAPQI binds to mitochondrial proteins and enhances the conversion of molecular oxygen into ROS, leading to depleted mitochondrial bioenergetics, onset of mitochondrial permeability transition, and triggering both necrotic and apoptotic cell death. In addition, nucleic acids, lipids, and membranes may be detrimentally affected by elevated levels of ROS [196]. SeNPs also efficiently reduce BUN, fibronectin, and collagen levels and enhance heat shock protein (HSP‐70) and longevity protein (SIRT1) levels in diabetic kidneys [197]. By regulating fibrotic pathways and protecting renal function, SeNPs can delay the progression of CKD. SeNPs can be surface functionalized with the antioxidant ligand 6‐hydroxy‐2,5,7,8‐tetramethylchroman‐2‐carboxylic acid (trolox), forming Se@Trolox [198]. Se@Trolox (∼100 nm, 10 and 20 μg/mL) blocked caspase‐mediated apoptosis arising from drug‐induced nephrotoxicity through inhibition of ROS‐mediated p53 phosphorylation. This was further confirmed by detection of chromatin condensation, DNA fragmentation, and PARP cleavage. The activation of caspase‐3, ‐8, and ‐9, induced by the nephrotoxic agent, was suppressed. The SeNPs also effectively protected normal kidney cells from cisplatin‐induced injury by scavenging ROS and regulating the AKT and MAPK pathways. Selenium has also been shown to upregulate anti‐apoptotic Bcl‐2 protein and downregulate pro‐apoptotic Bax protein [199]. Other types of SeNPs, such as those made using turmeric extract (Tur‐SeNPs, ∼15 nm, 0.5 mg/kg) lessened drug‐induced renal inflammation via reduction in expression of IL‐6 and tumor necrosis factor‐α (TNF‐α) [200]. Lycopene‐coated selenium nanoparticles (LYC‐SeNPs, ∼130 nm, 0.5 mg/kg), lowered rhabdomyolysis‐related renal biomarkers like neutrophil gelatinase‐associated lipocalin, serum urea, creatinine, TNF‐α, IL‐1β, and IL‐6, and downregulated nitric oxide synthase‐2 expression [201]. It is important to administer an appropriate dose of SeNPs in order to maximize therapeutic efficacy in treating kidney disease/injury and minimize side effects, as the use of selenium above 100 mg/kg can lead to interstitial hemorrhages and afferent blood vessel congestion [202].

## 7. Toxicological Issues of Inorganic Nanomaterials

Despite the promising therapeutic and diagnostic applications of inorganic nanomaterials for kidney diseases, their clinical translation faces significant limitations. One of the most critical concerns is that certain metal oxides, such as iron oxide (Fe_3_O_4_), can induce oxidative stress under physiological conditions. The dissolution of these materials releases free iron ions, which can participate in Fenton reactions and generate ROS, leading to cytotoxicity, DNA damage, and unwanted inflammation [203, 204]. Moreover, animal studies have shown that ultrasmall nanoparticles can trigger acute oxidative damage in multiple organs, resulting in significant toxicity even at moderate doses [205]. Another major challenge is their poor biodegradability in vivo, which leads to persistent accumulation in organs such as the liver, spleen, and kidneys. This raises concerns about chronic exposure, potential immunogenicity, and disruption of normal tissue homeostasis [206]. The biocompatibility and toxicity of IONPs are highly dependent on factors such as particle size, surface coating, and functionalization, all of which influence their biodistribution, degradation, and biological clearance [207]. These limitations underscore the need for careful surface engineering to improve nanoparticle stability and minimize ion release, along with dose optimization and long‐term toxicological assessments. They also reinforce the growing interest in hybrid or biodegradable systems, incorporating biocompatible materials such as plant‐derived antioxidants, to enhance therapeutic efficacy while reducing adverse effects.

## 8. Conclusions

Before implementing a therapeutic approach to treat kidney diseases in humans, it is essential to evaluate the administration route and design a synthesis strategy that ensures efficient delivery of active agents to the target site while minimizing adverse effects. Although the use of nanoparticles offers significant advantages, particularly in overcoming physiological barriers, their clinical application still faces important limitations. Current studies on inorganic nanoparticles derived from plant compounds are promising but present notable challenges, such as insufficient long‐term toxicity evaluations and limited data on biodistribution and renal targeting in complex biological systems. Moreover, most available evidence comes from small‐scale in vivo studies that do not fully replicate human renal pathophysiology. To overcome these shortcomings, future research should focus on establishing standardized and reproducible green synthesis protocols employing plant compounds with consistent bioactive properties, developing preclinical models that more accurately simulate human kidney function and disease progression, and designing nanoparticles with specific physicochemical characteristics that enable selective accumulation in renal tissue and sustained antioxidant release. In this context, inorganic nanoparticles functionalized with plant‐derived compounds hold great potential to enhance the kidney’s natural defense mechanisms against oxidative species. Their ability to cross glomerular filtration barriers and be functionalized with potent phytochemicals positions them as next‐generation therapeutic tools for renal protection, provided that the aforementioned challenges are adequately addressed.

## Conflicts of Interest

The authors declare no conflicts of interest.

## Funding

No funding was received for this manuscript.

## Data Availability

Data sharing is not applicable to this article as no datasets were generated or analyzed during the current study.
